# Characterization of Rabensburg Virus, a *Flavivirus* Closely Related to West Nile Virus of the Japanese Encephalitis Antigenic Group

**DOI:** 10.1371/journal.pone.0039387

**Published:** 2012-06-19

**Authors:** Matthew T. Aliota, Susan A. Jones, Alan P. Dupuis, Alexander T. Ciota, Zdenek Hubalek, Laura D. Kramer

**Affiliations:** 1 The Arbovirus Laboratories, New York State Department of Health Wadsworth Center, Slingerlands, New York, United States of America; 2 Institute of Vertebrate Biology, Academy of Sciences of the Czech Republic, Brno, Czech Republic; 3 Department of Biomedical Sciences, School of Public Health, State University of New York at Albany, Albany, New York, United States of America; Global Viral Forecasting Initiative, United States of America

## Abstract

Rabensburg virus (RABV), a *Flavivirus* with ∼76% nucleotide and 90% amino acid identity with representative members of lineage one and two West Nile virus (WNV), previously was isolated from *Culex pipiens* and *Aedes rossicus* mosquitoes in the Czech Republic, and phylogenetic and serologic analyses demonstrated that it was likely a new lineage of WNV. However, no direct link between RABV and human disease has been definitively established and the extent to which RABV utilizes the typical WNV transmission cycle is unknown. Herein, we evaluated vector competence and capacity for vertical transmission (VT) in *Cx. pipiens*; *in vitro* growth on avian, mammalian, and mosquito cells; and infectivity and viremia production in birds. RABV infection and replication only were detected on mosquito cells. Experimentally inoculated birds did not become infected. *Cx. pipiens* had poor peroral vector competence and a higher VT rate as compared to US-WNV in *Cx. pipiens.* As a result, we postulate that RABV is an intermediate between the mosquito-specific and horizontally transmitted flaviviruses.

## Introduction

West Nile virus (WNV), a member of the Japanese encephalitis virus (JEV) serogroup of the *Flavivirus* genus (family *Flaviviridae*) is grouped into two major genetic lineages. Lineage one WNV is widely distributed, occurring in Africa, Asia, Europe, Australia, and North and Central America [1]. In addition to causing West Nile fever in humans, Lineage one WNV is responsible for infection of the central nervous system in approximately 1% of cases, and may lead to a range of clinical outcomes including encephalitis, meningitis, acute flaccid paralysis and death [2]. Lineage one WNV is further divided into three sublineages: Lineage 1a is the most widely distributed, occurring in Africa, Europe, and the Americas; lineage 1b, also known as Kunjin virus occurs in Australia; lineage 1c occurs in India. Lineage two occurs in sub-Saharan Africa, where it is the cause of West Nile fever, a generally self-limiting illness that rarely progresses to severe disease in humans [3], but recently it has appeared in Europe (Hungary, Austria, Greece), causing avian, equine, and human outbreaks occasionally with encephalitis and fatalities. Recently, a third lineage of WNV called Rabensburg virus (RABV; prototype strain 97–103) has been proposed. RABV was isolated first from a pool of C*ulex pipiens* mosquitoes in South Moravia, Czech Republic in September of 1997 [4], again in the same location in 1999 [5], and more recently in 2007 it was isolated from a pool of *Aedes rossicus* [6]. RABV has 75–77% nucleotide identity and 89–90% amino acid identity with representative members of lineage one and two WNV [7], but RABV has shown partial antigenic heterogeneity with the Egyptian Eg101 topotype strain of WNV, a representative of lineage one WNV [8], in plaque-reduction cross-neutralization tests using homologous and heterologous antisera [5]. Charrel et al. (2003) initially defined membership in the species WNV to be <21% genetic distance [9], yet inclusion of RABV along with Russian and Indian strains of WNV would increase this criterion to over 25% divergence for some isolates [10], and would expand WNV into five distinct lineages [10]–[12].

RABV initially has been classified as a third lineage of WNV on the basis of genetic distance, but the biologic and antigenic differences between RABV and the WNV reference strain Eg-101 (lineage one) support the opinion that RABV is a novel *Flavivirus* [7], [10]. Although, serological evidence of WNV infection in febrile humans has been obtained in the Czech Republic [13] and this was concurrent to the initial isolation of RABV, no direct link between RABV and human disease has ever been established (i.e., no human isolate exists). The extent to which RABV utilizes the typical WNV transmission cycle is unclear. Consequently, in order to clarify the mechanism by which RABV is maintained in nature and, in turn, its ecological relationship to prototype WNV, we evaluated vector competence, both peroral and capacity for vertical transmission in *Cx. pipiens* mosquitoes; *in vitro* growth on mosquito, avian, and mammalian cell lines; and infectivity and viremia production in chickens and house sparrows. Our results suggest that RABV be considered an intermediate between the mosquito-specific flaviviruses and the horizontally transmitted flaviviruses in the JEV serogroup based on the genetic and biologic differences between it and WNV.

## Methods

### Ethics Statement

This study was carried out in strict accordance with the recommendations in the *Guide for the Care and Use of Laboratory Animals* of the National Institutes of Health. All animals and animal facilities were under the control of the Wadsworth Center Veterinary Science Program with oversight from the NYSDOH Division of Laboratory Operations and the protocol was approved by the Wadsworth Center Animal Care and Use Committee (Approval #09-412 and 09-355).

### Cells

African Green monkey kidney cells (Vero; ATCC #CCL-81) and baby hamster kidney cells (BHK; ATCC #CCL-10) were grown in minimal essential medium (MEM, Gibco, Carlsbad, CA) supplemented with 10% fetal bovine serum (FBS, Hyclone, Logan, UT), 2 mM L-glutamine, 1.5 g/l sodium bicarbonate, 100 U/ml of penicillin, and 100 µg/ml of streptomycin. *Aedes albopictus* mosquito cells, (C6/36, ATCC #CRL-1660) were maintained in MEM supplemented with 10% FBS, 2 mM L-glutamine, 1.5 g/l sodium bicarbonate, 0.1 mM non-essential amino acids, 100 U/ml of penicillin, and 100 µg/ml of streptomycin. Chicken embryo fibroblast cells (DF-1, ATCC #CRL-141) were cultured in Dulbecco’s modified Eagles’ medium (ATCC #30-2002) supplemented with 10% FBS, 2 mM L-glutamine, 100 U/ml of penicillin, 100 µg/ml of streptomycin. Human embryonic kidney cells (Hek293; ATCC #CRL-1573) were cultured in Dulbecco’s modified Eagles’ medium supplemented with 10% FBS, 2 mM L-glutamine, 1.0 mM sodium pyruvate, and 1.5 g/l sodium bicarbonate.

### Mosquitoes

All mosquitoes used in this study were maintained at the Arbovirus Laboratories, Wadsworth Center as described previously [14], [15]. Three different strains of *Culex pipiens* were used in the studies described: (1) *Cx. pipiens* (*CpUS)* mosquitoes originally collected during 2004 in Pennsylvania (courtesy of Michael Hutchinson, Pennsylvania State University, USA), that contain genetic signatures of form pipiens and form molestus. *Cx. pipiens* form pipiens are anautogenous bird-dependent feeders and *Cx. pipiens* form molestus are autogenous, breed in confined spaces, and are more likely to bite humans; (2) a pure line of *Cx. pipiens* form molestus (*CpEU*) from Europe (courtesy of Sander Koenraadt, Wageningen University, Netherlands), and (3) pure *Cx. pipiens* form molestus *(CxM)* from the United States (courtesy of Dina Fonseca, Rutgers University, USA). *Culex* genetics were verified as described [16], [17].

### Viruses

RABV isolate 97-103 (GenBank AY765264), originally isolated from *Cx. pipiens* in the Czech Republic, was obtained from Zdenek Hubalek (Institute of Vertebrate Biology, Academy of Sciences, Brno, Czech Republic). A virus stock was prepared by inoculation onto a confluent monolayer of C6/36 mosquito cells and a clarified harvest of the culture medium was collected after four days of incubation at 28°C. This stock was titered by fluorescent focus assay (FFA) on C6/36 cells (log_10_ 7.1 fluorescent foci units (FFU)/ml) [18]. WNV isolate WN02-1956 (GenBank AY590210) was isolated from the kidney of an American Crow collected in New York State and isolated on Vero cells, followed by a single round of amplification on C6/36 cells. The titer by plaque assay on Vero cells was log_10_8.6 plaque forming units (PFU)/ml.

### Identification of Virus Positive Samples

Virus positive experimental samples were confirmed via hemi-nested reverse transcriptase polymerase chain reaction (hnRT-PCR). Briefly, RNA was extracted from WNV and RABV using the MagMax™-96 Viral RNA isolation kit (Ambion) according to the manufacturer’s instructions using a Tecan Freedom EVO® robotic platform (Tecan, Mannedorf, Switzerland). Primer sets for hnRT-PCR targeted the NS5 region and were capable of detecting all members of the *Flaviviridae* [19]. For the first amplification, one-step RT-PCR (Qiagen) was conducted to produce an amplicon followed by hemi-nested amplification using the *Taq* PCR Core Kit (Qiagen).

### Viral Replication *in vitro* and Transfection of Hek293 Cells

Six-well plates containing confluent monolayers of DF-1, BHK, C6/36, Vero, or Hek293 cells were infected with virus (RABV97-103 or WN02-1956), in triplicate, at an MOI of 0.01 FFU per well. After one hour of adsorption at 28°C (C6/36), 37°C (mammalian), or 39°C (DF-1), the inoculum was removed, 3 ml of maintenance media was added to each well, and the plates were returned to the appropriate temperatures. Samples, consisting of 50 µl of media, were taken at 0.5–6 days post infection, diluted 1∶10 in culture media, and stored at −80°C.

Hek293 cells were transfected by electroporation as described by [20] with slight modifications. Instead of using RNA transcripts generated via *in vitro* transcription for the transfection, RABV RNA was extracted from infected C6/36 cell supernatant using the Qiagen RNeasy kit. Hek293 cells were transfected with 9 µg viral RABV RNA and medium was harvested from the transfected cells for seven days and subsequently used to infect new C6/36 cells. Cells were observed for cytopathic effect (CPE) and RNA was isolated from the infected C6/36 cells and used subsequently in a RT-PCR to verify RABV infection. C6/36 cells were transfected with 9 µg viral RABV RNA to serve as a positive technical control.

### Vector Competence

Vector competence was evaluated for *CpUS*, *CpEU,* and *CxM*. Infection, dissemination, and transmission rates were determined as described previously [21]. Briefly, mosquitoes were exposed to virus-infected bloodmeals using a Hemotek membrane feeding apparatus (Discovery Workshops, Accrington, UK). Bloodmeals consisted of defibrinated chicken blood (Rockland Inc.) and RABV from frozen stock, yielding a RABV concentration of 6.1 log_10_FFU/ml or a WNV concentration of 6.1 log_10_FFU/ml. Mosquitoes that fed to repletion were separated into 4.0 L cartons and maintained on 10% sucrose in an environmental chamber at 27°±2°C, 70%±10% relative humidity, and with a 16 hour photoperiod. All samples were screened by hnRT-PCR. Dissemination was indicated by virus-positive legs. Transmission was defined as release of infectious virus with salivary secretions, i.e., the ability to infect another host, and was indicated by virus-positive salivary secretions [14].

### Vertical Transmission

The capacity for RABV to be vertically transmitted in mosquitoes was assessed for *CxM, CpUS,* and *CpEU.* A finely pulled capillary microinjection needle was used to intrathoracically inject approximately 0.5 µl of undiluted RABV stock virus at a concentration of 7.1 log_10_ FFU/ml into seven- to ten-day-old female *CpUS, CpEU,* and *CxM* (n = 150). Females were allowed to mate for seven days prior to viral injection. Seven days post injection (PI) mosquitoes were exposed to an uninfected bloodmeal and allowed to oviposit. Third and fourth instar larvae were collected in pools of five and stored at −80°C until they were processed and assessed for viral infection via hnRT-PCR. The original adult, female mosquitoes were given two additional bloodmeals 14 and 21 days post initial blood feeding. This experiment was repeated twice with separate cohorts of mosquitoes.

### Infectivity and Viremia Production in Birds

Adult house sparrows (*Passer domesticus*) were caught in Albany County, New York, using mist nets under NYS Department of Environmental Conservation license no. 1236 and U.S. Fish and Wildlife permit no. NB035731-0. Birds were transported to the Arbovirus Laboratories, Wadsworth Center, treated for ectoparasites, and quarantined for two weeks. Following quarantine, birds were tested for antibodies to WNV via indirect enzyme-linked immunosorbent assay (ELISA) [22]. All seronegative birds then were moved into the BSL-3 laboratory and held for one week of acclimation. Six of the eight birds were inoculated with 10^4 ^PFU equivalents of RABV in 100 µl animal diluent (AD: 1% heat-inactivated FBS in Dulbecco’s PBS) by subcutaneous injection in the cervical region. The two remaining birds were inoculated with AD alone to serve as experimental controls. Blood samples were taken from half of the birds 1, 3, 5 d PI and from the other half of the birds on 2, 4, 6 d PI as described previously [23]. At 21 d PI, a 0.1 ml blood sample again was taken to measure RABV antibody status, and birds then were euthanized by overdose of pentobarbital (15 mg/kg).

Pathogen-free chicken eggs (*Gallus gallus*) were obtained from Sunrise Farms (Catskill, NY, USA) and hatched at the Arbovirus Laboratories. Chickens were separated into two experimental groups (five/group) and housed in metal cages with individual light sources and daily fresh food, water, and resting pads. One- to two-day-old chickens were inoculated subcutaneously with approximately 10^4^ PFU equivalents of RABV (Group 1) or 10^7^ PFU equivalents of RABV (Group 2). Two additional chickens were inoculated subcutaneously with AD alone to serve as experimental controls. All three groups were housed separately in adjacent cages and monitored daily for signs of illness. Chickens were bled from the brachial vein and 50–100 µl blood was collected by capillary action in glass capillary tubes on days 1–5 PI, diluted 1∶10 in BA-1, and stored at −80°C until tested. At day 14 PI, blood was collected to determine antibody status, and chickens were euthanized by overdose of pentobarbital (15 mg/kg).

### Statistical Analysis

Infection, dissemination, and transmission rates were analyzed using an Exact unconditional test [24]. Vertical transmission rates were expressed as the maximum likelihood estimation of infection rates (MLE-IR), which is defined as the infection rate most likely observed given the testing results and an assumed probabilistic model (i.e., binomial distribution of infected individuals in a positive pool) [25].

## Results

### Viral Replication *in vitro* and *in vivo*

**Figure 1:**
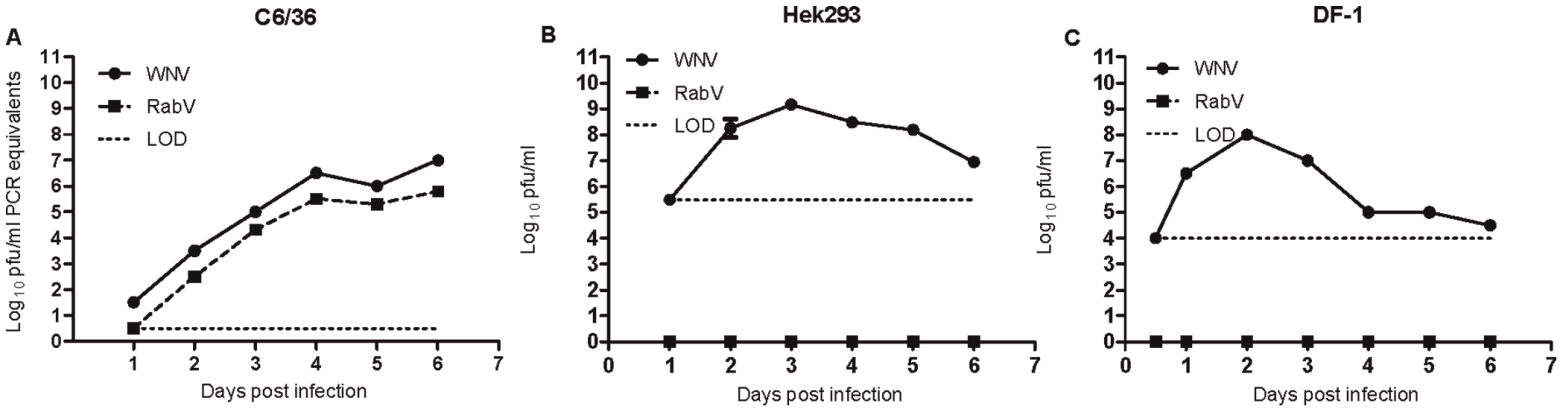
Rabensburg virus growth on mosquito, human, and avian cells. Data points represent means of three replicates at each time point +/− standard deviation. LOD, limit of detection; WNV, West Nile virus strain WN02-1956; RABV, Rabensburg virus strain 97–103; C6/36, *Aedes albopictus* cells; Hek293, human kidney cells; DF-1, chicken cells; pfu, plaque forming units. **A.)** Mosquito Cells. **B.)** Human Cells. **C.)** Avian Cells.

To begin to understand which vertebrate hosts maintain RABV in nature and its ecological relationship to prototype WNV, growth kinetics of both viruses were assessed on five cell lines: C6/36 (mosquito), Vero (monkey), hamster (BHK), human (Hek293), and avian (DF-1). C6/36 cells inoculated with RABV97-103 displayed overt CPE, and viral growth on mosquito cells that were inoculated with either RABV97-103 or WNV-WN02-1956 did not differ significantly between the two viruses over the course of infection (Figure 1A). In contrast, viral growth on vertebrate cell culture did differ significantly between RABV and WNV, i.e., no replication of RABV was observed on BHK, DF-1, Hek293, or Vero cells (see Figure 1B and 1C for representative growth curves). In contrast, Hek293 cells transfected via electroporation with RABV RNA did produce infectious virus as confirmed by the fact that C6/36 cells inoculated with supernatant from RABV RNA-transfected Hek293 cultures exhibited an overt CPE, whereas C6/36 cells inoculated with supernatant from mock-transfected Hek293 cultures appeared normal. Our data thus indicate that the inability of RABV to replicate in human cells occurs at the point of entry, as infectious RABV RNA was able to replicate in human cells following transfection. In addition to *in vitro* growth kinetics, infectivity and viremia production were assessed *in vivo* in experimentally inoculated birds (house sparrows and chickens). Viremia was not detected in either house sparrows or chickens over the course of experimentation, nor was antibody to RABV detected at 14 d PI (data not shown).

### Vector Competence

It is a well-established fact that mosquitoes in the genus *Culex* are competent vectors of WNV, and previous work in our laboratory has demonstrated that our colonized *Cx. pipiens* (*CpUS*) are competent vectors of WNV [14], [26]. In contrast, the ability of RABV to infect, disseminate, and be transmitted by *Culex* mosquitoes is unknown; therefore, we assessed vector competence for RABV in *CpEU* and *CpUS*. Mosquitoes that ingested blood containing RABV were assayed for viral infection, dissemination, and transmission and both mosquito strains displayed poor peroral vector competence as compared to the same mosquitoes infected with WNV-WN02-1956 (Table 1 and 2). At 14 d PI, the transmission rate in RABV-infected *CpEU* was 24% and in RABV-infected *CpUS* it was 12%. A poor transmission rate (i.e., 12%) also was observed at 21 d PI in *CxM* infected with RABV (data not shown). Additionally, infection, dissemination, and transmission were assessed in *CpUS* and *CpEU* for both RABV and US-WNV, and there was a significant reduction (Exact Unconditional Test, p = 0.028) in RABV-positive salivary secretions as compared to US-WNV 14 d post blood feeding in *CpEU* (Table 1). The same general trend (i.e., a reduced transmission rate in mosquitoes infected with RABV) was observed for *CpUS* infected with US-WNV or RABV but the data were not statistically significant (Table 2). The low transmission rates observed in *CpUS* infected with US-WNV may be related to bloodmeal titer (10^6^ pfu/mL), i.e., mosquito vectors do not become infected efficiently when WNV bloodmeal titers are <10^5^ pfu/mL, and higher viral titers in the bloodmeal increase the probability of mosquito infection [27]. Based on these results, in conjunction with RABV growth kinetics, we postulated that RABV might be a mosquito-specific virus, and if mosquitoes poorly transmitted the virus then RABV must be maintained in nature by some other method, i.e., vertical transmission (VT).

**Table 1:** Vector competence of European-*Culex pipiens* form molestus following peroral infection.*

	Days post-feeding					
Virus	7			14		
	I	D	T	I	D	T
RABV	48 (n = 40)	37 (n = 19)	0	74 (n = 34)	96 (n = 25)	24 (n = 25)
WN02-1956	ND	ND	ND	62 (n = 34)	100(n = 21)	57 (n = 21)
*p* value†	ND	ND	ND	0.366	1.00	0.028

* I, % infected; D, % disseminated (of infected); T, % transmitting (of infected); ND, no data.

† Calculated using an exact unconditional test.

**Table 2:** Vector competence of U.S.-*Culex pipiens* following peroral infection.*

	Days post-feeding					
Virus	7			14		
	I	D	T	I	D	T
RABV	43 (n = 40)	47 (n = 17)	6 (n = 17)	65 (n = 40)	42 (n = 26)	12 (n = 26)
WN02-1956	30 (n = 40)	42 (n = 12)	17 (n = 12)	55 (n = 40)	41 (n = 22)	18 (n = 22)
p value†	0.272	1.00	0.500	0.434	1.00	1.00

* I, % infected; D, % disseminated (of infected); T, % transmitting (of infected).

† Calculated using an exact unconditional test.

### Vertical Transmission

To ascertain if VT could be responsible for the maintenance of RABV in mosquito populations, we conducted experiments using *CpEU, CpUS,* and *CxM* to determine the capacity of RABV to be vertically transmitted. Mosquitoes were infected with RABV via intrathoracic inoculation of virus, because if RABV were a mosquito-specific virus then, in nature, mosquitoes would never acquire the virus via blood feeding. Following inoculation, the virus was allowed to disseminate throughout the mosquito body cavity. Larvae were collected, screened via hnRT-PCR for RABV infection, and VT was observed in all three strains of mosquitoes (Table 3). *CpEU* had the highest overall MLE-IR of 41.5, followed by *CpUS* with 32.6 and *CxM* with 21.3. VT was observed in two independent experiments utilizing different cohorts of mosquitoes for testing for *CpUS* and *CxM* and for *CpEU,* VT was observed in one out of two replicate experiments.

**Table 3:** Vertical transmission of Rabensburg virus in three strains of *Culex pipiens.* *

Mosquito Strain	Oviposition dpi	No. of pools tested†	MLE [positive pools/total]
*CpUS*			
OP1 Larvae	7	151	46.5 [32/151]
OP2 Larvae	14	69	15.0 [5/69]
OP3 Larvae	21	22	0.00 [0/22]
		MLE Combined	32.6 [37/242]
*CpEU*			
OP1 Larvae	7	43	40.3 [8/43]
OP2 Larvae	14	23	47.8 [5/23]
OP3 Larvae	21	2	0.00 [0/2]
		MLE Combined	41.5 [13/68]
*CxM*			
OP1 Larvae	7	35	5.78 [1/35]
OP2 Larvae	14	9	49.0 [2/9]
OP3 Larvae	21	5	97.1 [2/5]
		MLE Combined	21.3 [5/49]

* MLE, maximum likelihood estimation of infection rates; *CpUS*, a United States strain of *Culex pipiens* that contain genetic signatures of both form pipiens and form molestus; *CpEU,* a European strain of *Culex pipiens* form molestus; *CxM*, a United States strain of *Culex pipiens* form molestus; OP, oviposition; dpi, days post infection; Vertical transmission of RABV was observed in two independent experiments for *CpUS* and *CxM* and in one out of two independent replicates for *CpEU.* All surviving parental females were pooled in groups of five, screened for viral infection, and all pools were RABV positive.

† pool size = 5.

### Sequence Analysis

Cell fusing agent virus (CFAV), Culex flavivirus, and several other recently discovered members of the genus *Flavivirus* have no known vertebrate host and are thought to infect mosquitoes only. Firth et al. (2010) identified a ∼300 codon gene (designated *fifo*) conserved throughout the mosquito-specific flaviviruses that overlaps the NS2A and NS2B coding sequences [28]. We analyzed the RABV genome to identify whether *fifo* was present, because the results from our viral replication, VT, and vector competence experiments suggested that RABV was a mosquito-specific virus. However, sequence analysis of the RABV genome revealed a 45 codon open reading frame (ORF) (designated *foo*) that is conserved among the JEV serogroup flaviviruses (e.g., *Murray Valley encephalitis virus*, *St. Louis encephalitis virus*, WNV) [29], [30]. RABV shares only ∼48% nucleotide identity with representative members of the mosquito-specific flaviviruses (vs. >70% nucleotide identity with representative members of the JEV serogroup) suggesting that perhaps RABV is a link between the mosquito-specific flaviviruses and the JEV complex of viruses. Further analysis of the RABV genome revealed the absence of an N-linked glycosylation motif at NS1 site 207. RABV contains N-linked glycosylation sites at E_154,_ NS1_130_, and NS1_175_. WNV strains may contain either one or no N-linked glycosylation motif in the E protein (E_154_), but all WNV strains contain three highly conserved NS1 sites (NS1_130_, NS1_175_, NS1_207_). In fact, all members of the JE serogroup, with the exception of JEV contain all three NS1 glycosylation motifs [31]–[34].

## Discussion

In sum, we postulate that RABV is an intermediate between the mosquito-specific flaviviruses and the JEV complex of viruses. This hypothesis is supported by the fact that unpassaged RABV97-103 did not infect mammalian and avian cell culture, house sparrows or chickens, but the virus efficiently infected mosquito cells. It should be noted that replication and CPE have been observed on a clone of standard Vero cells (E6) and *Xenopus laevis* frog cells (XTC-2); however, passage via intracranial inoculation of suckling mice occasionally has been required for growth on Vero E6 cells. A third isolate (RABV06-222; GenBank:GQ421359) produced CPE after original inoculation of mosquito suspension on Vero E6 cells [4], [6], [7]. It also has been shown that RABV exhibited a considerably lower virulence in suckling mice as compared to WNV-Eg-101. RABV97-103 killed all suckling mice after intracerebral inoculation, but following intraperitoneal inoculation it killed only one third of suckling mice and the average survival time was 11 days. Adult mice survived even when given the virus intracerebrally [5]. Interestingly, RABV06-222 was found to kill newborn mice after intracerebral, intraperitoneal, or subcutaneous administration [6].

In addition, mosquitoes within the *Culex pipiens* complex supported replication of RABV but displayed poor peroral vector competence for this virus as compared to WNV-WN02-1956, and the same mosquitoes vertically transmitted the virus at a much higher rate than what has been reported for wild type WNV in US-*Cx. pipiens.* In 2002, Dohm et al. demonstrated that U.S.-*Cx. pipiens* could vertically transmit WNV, albeit at a much lower rate than what was observed in this study, i.e., they calculated an overall minimal filial infection rate of 1.8/1000 (or an approximated MLE-IR of 1.86) [35] versus the calculated overall MLE-IR of 32.6 in our *CpUS,* 21.3 in *CxM*, and 41.5 in *CpEU*. We also have observed VT of wild type WNV in *CpUS* at a MLE-IR of 2.9 following an infectious bloodmeal (Aliota et al., unpublished). In addition, Turell et al. (2001) demonstrated VT of WNV in laboratory *Cx. pipiens* [36], and Miller et al. (2000) identified male, WNV-infected *Culex univittatus* in Kenya [37] indicating that VT of WNV does occur in nature and probably is responsible for seasonal maintenance of the virus in more temperate regions [38].

The “intermediate” hypothesis is supported further by the fact that it recently has been shown that the filial infection rate of vertically transmitted Culex flavivirus, a virus that only infects mosquitoes, was 97.4% [39], and by the fact that *Aedes albopictus* exposed to a CFAV-infected bloodmeal did not become infected with CFAV (Aliota et al., unpublished). Clearly, some of our mosquitoes were capable of transmitting RABV, and it has not yet been excluded that RABV might circulate and be amplified in certain vertebrates, e.g., amphibians [4], [6], [7]. Additionally, sequence analysis of the RABV genome revealed the presence of an ORF that is conserved among the JEV complex of viruses, and it revealed the absence of an N-linked glycosylation motif at NS1 site 207. This was interesting because WNV strains may contain either one or no N-linked glycosylation motif in the E protein (E_154_), but all WNV strains contain three highly conserved NS1 sites (NS1_130_, NS1_175_, NS1_207_). In fact, all members of the JE serogroup, with the exception of JEV contain all three glycosylation motifs [31]–[34]. N-linked glycosylation plays an important role in both the assembly and the infectivity of many viruses (e.g., [40]–[43]). Studies have demonstrated that non-glycosylated NS1 can influence neuroinvasiveness and impair replication for dengue and yellow fever, and deglycosylation of both E and NS1 proteins of WNV completely attenuated neuroinvasiveness and induced protective immunity in the murine model with low doses of virus [44].

The majority of previous studies on flavivirus evolution have suggested that arthropod-mediated transmission is a derived trait within the genus, with the ancestral condition being non-vector transmission [45]. Therefore, it would be appropriate to assume that at least some of the current group of horizontally transmitted flaviviruses evolved from mosquito-specific viruses. Recently, there has been an upsurge in the discovery of ‘insect-specific’ flaviviruses and/or their related sequences in natural mosquito populations [46]. And considering the high genetic plasticity associated with flaviviruses, it is reasonable to assume that some of these mosquito-specific viruses may emerge and adapt to new host environments, i.e., humans or other vertebrates. Timely recognition of emerging infections depends on an understanding of all of the factors involved in the maintenance and spread of an infectious organism. Future studies utilizing RABV could provide significant insight into the determinants of flavivirus attenuation in vertebrates and vertical transmission in mosquitoes. Specifically, RABV could be used to elucidate genetic changes that facilitate host switching, which may lead to new vertebrate pathogens or new transmission pathways. Additionally, it could increase our understanding of the link between the ‘insect-specific’ flaviviruses and those that are transmitted between mosquitoes and vertebrates, further clarifying the evolution of flaviviruses.
